# Greatly Enhanced Thermoelectric Performance of Flexible Cu_2−x_S Composite Film on Nylon by Se Doping

**DOI:** 10.3390/nano14110950

**Published:** 2024-05-28

**Authors:** Xinru Zuo, Xiaowen Han, Zixing Wang, Ying Liu, Jiajia Li, Mingcheng Zhang, Changjun Huang, Kefeng Cai

**Affiliations:** Key Laboratory of Advanced Civil Engineering Materials, Ministry of Education, School of Materials Science & Engineering, Tongji University, Shanghai 201804, China

**Keywords:** Cu_2_S, thermoelectric, doping, flexible, film

## Abstract

In this work, flexible Cu_2−x_S films on nylon membranes are prepared by combining a simple hydrothermal synthesis and vacuum filtration followed by hot pressing. The films consist of Cu_2_S and Cu_1.96_S two phases with grain sizes from nano to submicron. Doping Se on the S site not only increases the Cu_1.96_S content in the Cu_2−x_S to increase carrier concentration but also modifies electronic structure, thereby greatly improves the electrical properties of the Cu_2−x_S. Specifically, an optimal composite film with a nominal composition of Cu_2−x_S_0.98_Se_0.02_ exhibits a high power factor of ~150.1 μW m^−1^ K^−2^ at 300 K, which increases by ~138% compared to that of the pristine Cu_2−x_S film. Meanwhile, the composite film shows outstanding flexibility (~97.2% of the original electrical conductivity is maintained after 1500 bending cycles with a bending radius of 4 mm). A four-leg flexible thermoelectric (TE) generator assembled with the optimal film generates a maximum power of 329.6 nW (corresponding power density of 1.70 W m^−2^) at a temperature difference of 31.1 K. This work provides a simple route to the preparation of high TE performance Cu_2−x_S-based films.

## 1. Introduction

Flexible thermoelectrics (TEs) can be used in self-powered technologies and hold promise in wearable sensors and electronics for health and environment monitoring, which has attracted more and more attention in recent years [1,2,3]. Flexible TE generators (f-TEGs), which are easily bent to well fit the curved skin surface of the human body, can utilize temperature difference (Δ*T*) between the human body and environment based on the Seebeck effect [4]. In addition, this kind of generator has advantages that traditional generators do not have: no moving parts, no noise, no pollution, and maintenance-free. In the past decade, a variety of flexible TE materials has been developed, some of which have been assembled into f-TEGs that have demonstrated the ability to generate electricity at the nW or even μW level and to power some body sensors [5,6]. To evaluate the energy conversion efficiency of a material, the dimensionless figure of merit called *ZT* is introduced and quantified by *ZT = α^2^σT/κ*, where *α* is the Seebeck coefficient, *σ* is the electrical conductivity, *κ* is thermal conductivity, and *T* is absolute temperature [7]. Usually, *α^2^σ*, called power factor (PF), is used to evaluate the performance of TE films [8]. According to the definition, a high PF and a low *κ* are indispensable for a good TE material.

Flexible TE films need to have both good flexibility and TE performance. Depending on the presence or absence of a substrate, they can be categorized into self-supporting films or films on flexible substrates [9]. The former mainly focuses on conducting polymers and their composite materials [10,11], which possess the benefits of low weight, inherent low κ, and intrinsically high flexibility [12], but with less favorable TE performance compared to inorganic TE materials. The latter, using flexible substrates to support inorganic materials, can take advantage of the high PF of inorganic TE materials and the high flexibility of organic materials [13]. Bi_2_Te_3_ is the best TE material near room temperature (RT). Bi_0.4_Sb_1.6_Te_3_/Te particles were deposited on a Kapton surface by screen printing and then heat treated, and the flexible BiSbTe-based film exhibited an ultrahigh PF of ~3000 μW m^−1^ K^−2^ at RT [14]. In addition, Ag_2_Se is a promising alternative to Bi_2_Te_3_, with a PF up to 3520 μW m^−1^ K^−2^ for Ag_2_Se bulks at 300 K [15]. In 2019, our group prepared the flexible Ag_2_Se film on a nylon membrane by vacuum-assisted filtration and hot pressing. The film demonstrated a PF of 987 μW m^−1^ K^−2^ and good flexibility at RT [16]. Since then, a lot of investigations have been conducted on flexible Ag_2_Se film [17,18,19,20,21]. Recently, Liu et al. [22] prepared Ag/Ag_2_Se composite film on a nylon membrane by adjusting the nominal ratios of Ag/Se based on a one-pot method, and the film showed a high PF of ~2275 μW m^−1^ K^−2^ at RT. Nevertheless, low reserves, high cost, and toxicity for the elements Te and Se limit the application of Bi_2_Te_3_ and Ag_2_Se.

Compared with Te and Se, S has the advantage of abundant reserve, low toxicity, and low cost. Among TE sulfides, Cu_2−x_S (0 ≤ x ≤ 0.25), which is a candidate for “phonon-liquid electron-crystal”, possesses a low lattice thermal conductivity [23,24] and has become a hot research topic in the TE community. Cu_2−x_S shows very complicated low temperature crystal structures, and the TE performance is sensitive to x (Cu deficiency). With the increase in x, Cu_2−x_S (such as Cu_1.96_S, Cu_1.8_S) demonstrates an increased *σ* and a decreased *α*. Typically, the stoichiometric Cu_2_S has a high α of ~300 μV K^−1^ but a low *σ* (≤10 S cm^−1^) at RT, which limits its TE application. In order to improve the *σ* of Cu_2_S, many efforts have been made by doping and phase tuning. Wang et al. [25] optimized the hole concentrations by tuning the chemical bonding in Mn-doped Cu_2_S. The *σ* of optimal sample increased from ~50 to ~400 S cm^−1^ at 323 K. However, the sample with an optimal *σ* exhibited a PF of ~100 μW m^−1^ K^−2^ at 323 K, which was not significantly enhanced due to the large reduction in *α* compared to the pristine sample. Yao et al. [26] prepared Cu_2_S_1−x_Se_x_ bulks by mechanical alloying and then spark plasma sintering (SPS), and they found that Se doping could increase carrier concentrations by narrowing the band gap. With *α* maintained at a proper value (above 100 μV K^−1^), Cu_2_S_0.85_Se_0.15_ showed an enhanced PF of 125 μW m^−1^ K^−2^ at 323 K. Phase tuning of Cu_2−x_S has also attracted much attention recently. It is of interest to note that Cu_1.96_S possesses higher *σ* than Cu_2_S [27]. If the fraction of Cu_1.96_S in Cu_2−x_S can be adjusted, Cu_2−x_S would obtain an enhanced *σ* compared to Cu_2_S. Yang et al. [28] achieved the modulation of Cu_1.96_S content in Cu_2−x_S composite by adjusting the molar ratio of raw materials. Benefiting from the introduction of Cu_1.96_S, the *σ* was increased from ~10 S cm^−1^ to 230 S cm^−1^ at 300 K. Nevertheless, the *α* dropped to ~50 μV K^−1^ at RT, resulting in the PF of Cu_2−x_S composite at 300 K (~50 μW m^−1^ K^−2^) lower than that of pristine Cu_2_S. Recently, Yue et al. [29] synthesized a Cu_2−x_S micro/nanocomposite by a hydrothermal method combined with SPS. And the *σ* reached ~1050 S cm^−1^ at 323 K due to the synergistic effect of the increased Cu_1.96_S content and the introduction of nanostructures. As a consequence, the micro/nano Cu_2−x_S bulks presented a large PF of ~300 μW m^−1^ K^−2^ at 323 K.

In this work, we synthesized a series of Se-doped Cu_2−x_S powders based on a simple hydrothermal method and prepared Cu_2−x_S_1−y_Se_y_ films on a nylon membrane by vacuum-assisted filtration and hot pressing. By adjusting the nominal content of Se, the electrical properties of Cu_2−x_S films were improved. An optimized PF of 150.1 μW m^−1^ K^−2^ was obtained from the Cu_2−x_S_0.98_Se_0.02_ film at RT. The flexibility of the film and output performance of an assembled four-leg f-TEG were studied.

## 2. Materials and Methods

Cu_2−x_S_1−y_Se_y_ (y = 0,0.01,0.02,0.03) powders were synthesized by a facile hydrothermal method based on our recent work [30]. Subsequently, the dried powders were dispersed in ethanol, then deposited on the nylon membranes by vacuum-assisted filtration, and finally hot pressed at 270 °C and 1 MPa for 30 min. It should be noted that the Se content (y) is a nominal one. The preparation process of the Cu_2−x_S_1−y_Se_y_ composite film on a nylon membrane is shown in Figure S1 in Supplementary Materials. Details of raw materials and experiments are given in Note S1 of Supplementary Materials. The assembly process of the f-TEG is described in Note S2. The details of various characterizations and related measurements for the films and f-TEG are given in Note S3.

## 3. Results and Discussion

All the Cu_2−x_S_1−y_Se_y_ (y = 0,0.01,0.02,0.03) powders have similar diffraction patterns, corresponding well to tetragonal Cu_2_S (T-Cu_2_S, PDF#72-1072) and Cu_1.96_S (T-Cu_1.96_S, PDF#29-0578), shown in Figure S2a. When the doping content (y) increases to 3%, additional peaks that belong to Cu_1.8_S (PDF#24-0061) appear. Since Cu_2−x_S has different crystal structures, it is difficult to synthesize a pure Cu_2_S phase by the hydrothermal method [31]. The Cu_2−x_S_1−y_Se_y_ powders consist of particles with sizes between 100 and 500 nm (see Figure S2b).

**Figure 1:**
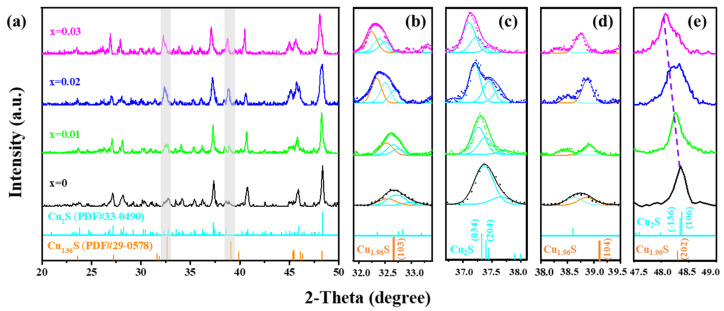
XRD patterns of the Cu_2−x_S_1−y_Se_y_ (y = 0,0.01,0.02,0.03) films with 2θ range of (**a**) 20°–50° (the peaks marked with the light gray box in Figure 1a correspond to the two strongest peaks of Cu_1.96_S), (**b**) 32.0°–33.3° (the orange line corresponds to the (103) plane diffraction of Cu_1.96_S, and the cyan lines are indexed to Cu_2_S), (**c**) 36.7°–38.2° (the cyan lines correspond to the (034) and (204) plane diffractions of Cu_2_S, respectively), (**d**) 38.0°–39.0° (the orange line corresponds to the (104) plane diffraction of Cu_1.96_S, and the cyan line is indexed to Cu_2_S), and (**e**) 47.5°–49.0° (the strongest peak for the Cu_2−x_S_1−y_Se_y_ films). The dots and lines in (**b**–**d**) are the raw data and fitted data, respectively.

Figure 1 displays the XRD patterns of the Cu_2−x_S_1−y_Se_y_ (y = 0,0.01,0.02,0.03) films. As shown in Figure 1a, the diffraction peaks of all films can be indexed to monoclinic Cu_2_S (M-Cu_2_S, PDF#33-0390) and T-Cu_1.96_S. The T-Cu_2_S converts to the M-Cu_2_S during hot pressing, which is consistent with our previous report [30]. With the increase in Se content, the peaks corresponding to the T-Cu_1.96_S (marked with the light gray box) become stronger, indicating that the fraction of the T-Cu_1.96_S increases. To discuss the effect of Se doping on the content of the T-Cu_1.96_S, we calculated the ratio of the T-Cu_1.96_S and M-Cu_2_S (abbreviated as T:M) according to the following equation [32]:

(1) $$T:M=\frac{I_{T-{Cu}_{1.96}S}^{\left(103\right)}+I_{T-{Cu}_{1.96}S}^{\left(104\right)}}{I_{M-{Cu}_{2}S}^{\left(034\right)}+I_{M-{Cu}_{2}S}^{\left(204\right)}}$$

where *I* is the integral intensity of different diffractions. The ratio of the above two phases can be quantified in the 2θ = 32–39.5° region. The peaks corresponding to (103) and (104) planes are the strongest in the T-Cu_1.96_S. The planes of (034) and (204) belong to the M-Cu_2_S. These peaks are all highlighted in Figure 1b–d, where the orange and cyan fitting lines correspond to the diffractions of T-Cu_1.96_S and M-Cu_2_S, respectively. Herein, the results based on Equation (1) for all samples are listed in Table 1, and it can be seen that the value of T:M increases from 0.39:1 at y = 0 to 0.85:1 at y = 0.03, indicating that the T-Cu_1.96_S content increases with Se doping. Figure 1e shows that the strongest peak of the films shifts to lower angles with x increasing from 0 to 0.03, which is due to Se^2−^ having a larger ionic radius (1.98 Å) than S^2−^(1.84 Å). It is evident that Se doping will lead to the expansion of the Cu_2_S lattice.

In order to obtain a better understanding of the composition and elemental valence of the Cu_2−x_S_1−y_Se_y_ films, XPS measurements were carried out for the Cu_2−x_S_0.98_Se_0.02_ film (see Figure S3). The high-resolution spectrum of Cu 2p demonstrates two strong peaks at 932.7, 952.5 eV corresponding to Cu^+^ [33]. The weak split peaks at about 934.8 eV (Cu 2p_3/2_), 954.9 eV (Cu 2p_1/2_), and the satellite peaks are attributed to Cu^2+^, which is due to the presence of Cu_1.96_S [34,35]. Two characteristic peaks of S 2p_3/2_ and S 2p_1/2_ are located at 161.8 and 163.0 eV, respectively. Deconvolution of the Se 3d peaks at 54.0 and 55.1 eV suggests that Se has been successfully doped into the Cu_2−x_S films as a divalent ion [36,37]. In particular, the XPS spectra of Cu 2p (Figure S4) show that the binding energy of the peaks shifts toward lower values and the proportion of Cu^2+^ increases with increasing y, indicating the formation of more Cu_1.96_S [38], which agrees well with our calculation results in Table 1.

**Figure 2:**
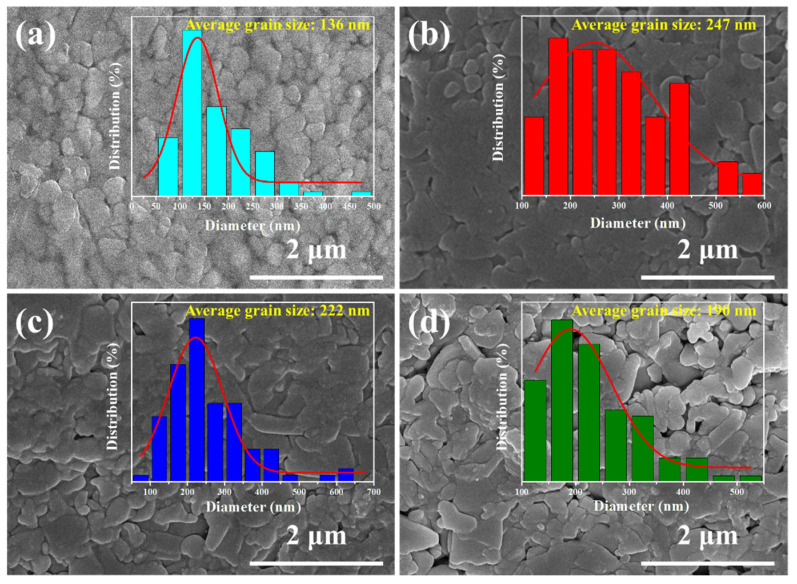
SEM images of the Cu_2−x_S_1−y_Se_y_ (y = 0.01,0.02,0.03) films: (**a**) y = 0, (**b**) y = 0.01, (**c**) y = 0.02, (**d**) y = 0.03. The insets in (**a**–**d**) show the distribution of grain size, and Gauss fit (the red line) enveloping grain size distributions is shown to guide the eye.

Figure 2 shows the SEM images for the Cu_2−x_S_1−y_Se_y_ films. The grain boundaries are obscure, which suggests that the films have been sintered only to some extent. The average grain size for Se-doped films lies at 190–250 nm, which is larger than that of the Cu_2−x_S film (~136 nm). It is consistent with the phenomena observed in Se-doped Cu_2_S bulk [26]. The grain growth is facilitated by the large diffusion rate and small diffusion activation energy of Se in the substitution solid solution [39]. The distribution of elements in the Se-doped Cu_2−x_S film is further examined by elemental mapping shown in Figure S5. The elements of Cu, S, and Se are evenly distributed.

**Figure 3:**
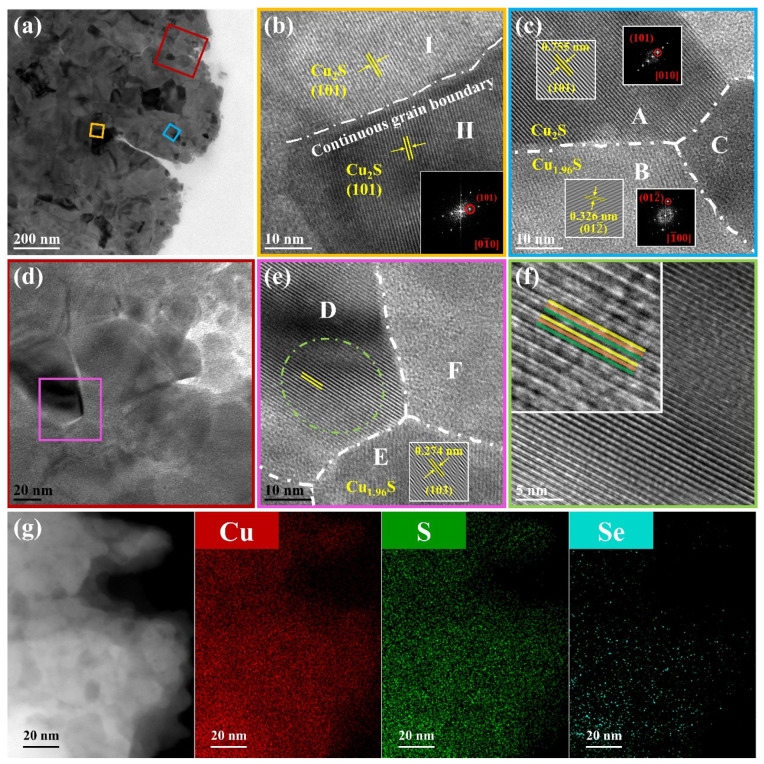
TEM analysis of the Cu_2−x_S_1−y_Se_y_ film (y = 0.02): (**a**) Typical TEM image. (**b**–**d**) Enlarged images of the orange, blue, and red squares marked in (**a**), respectively. Insets are the fast Fourier transform (FFT) and inverse fast Fourier transform (IFFT) images of corresponding grains. (**e**) Enlarged image of the pink square marked in (**d**), and the inset is the IFFT image of grain E. (**f**) Enlarged image of the green dashed circle marked in (**e**) for grain D. The inset is partial magnification of (**f**). (**g**) HAADF image and corresponding Cu, S, and Se maps, respectively.

As an example, the internal microstructure of the Cu_2−x_S_0.98_Se_0.02_ film was observed by TEM, and the results are presented in Figure 3. As Figure 3a,b shows, the film contains submicron grains (above 100 nm) and significant number of nanograins with size of 20–100 nm. The size of these nanograins is smaller than that of the Cu_2−x_S powder (see Figure S2), indicating the powder underwent a melting and recrystallization process. A clear grain boundary (GB) can be seen between two grains in the high-resolution TEM (HRTEM) (Figure 3b). The two grains form a continuous GB, corresponding to the (101) plane of Cu_2_S, with a misalignment between two (101) planes of about 15°. The continuous GB favors the carrier transport. Figure 3c is an enlarged image of the blue square marked in Figure 3a, showing a typical triangular GB with different orientations of the zone axis. And the three grains (grains A, B, and C) are well bonded. The lattice spacing of grain A and grain B is about 0.755 and 0.326 nm, corresponding to the (101) plane of Cu_2_S and the (01$\bar{2}$) plane of Cu_1.96_S, respectively, which also indicates the coexistence of Cu_2_S and Cu_1.96_S in the Cu_2−x_S_0.98_Se_0.02_ film. Moreover, Figure 3d is an enlarged TEM image of the area marked by the red square in Figure 3a, indicating that the film contains nanograins. Figure 3e is an enlarged image of the pink square marked in Figure 3d, which contains three grains (grains D, E, and F) forming a triangle boundary. It can be seen from Figure 3f that grain D looks to have a very wide lattice spacing. However, the HRTEM image (inset of Figure 3f) reveals that there exist two additional lattice planes marked by green and brown in between the planes marked by yellow. The lattice spacing of grain E is ~0.274 nm, corresponding to the (103) plane of Cu_1.96_S. A typical high-angle annular dark field (HAADF) image and corresponding EDS images are shown in Figure 3g, which indicates that Cu, S, and Se are homogeneously distributed in the Cu_2−x_S_1−y_Se_y_ film.

**Figure 4:**
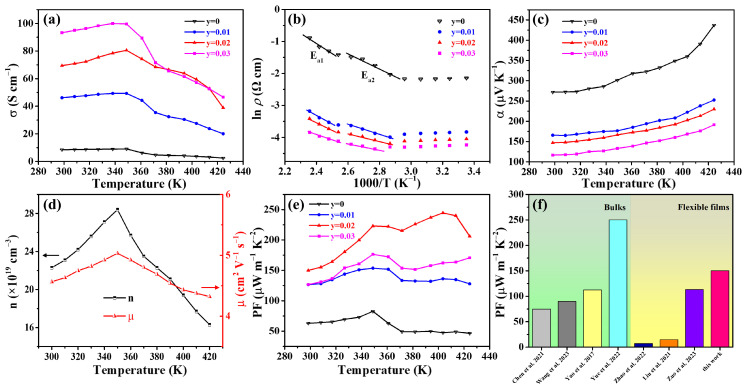
TE parameters of the Cu_2−x_S_1−y_Se_y_ (y = 0,0.01,0.02,0.03) films: (**a**) temperature dependence of σ, (**b**) plot of ln *ρ* vs. 1/T, (**c**) temperature dependence of *α*, (**d**) temperature dependence of *n* and *μ* for the Cu_2−x_S_0.98_Se_0.02_ film, (**e**) temperature dependence of PF, (**f**) comparison of PF between this work and other reported Cu_2_S-based bulks [25,26,29,43] and flexible films [30,44,45] at room temperature.

The temperature dependence TE parameters for the Cu_2−x_S_1−y_Se_y_ (y = 0,0.01,0.02,0.03) films are presented in Figure 4. As the temperature rises, the *σ* for all samples exhibits the same trend with a turning point at about 350 K in Figure 4a, which corresponds to the phase transition of Cu_2−x_S. With the increase in Se content, the *σ* at RT enhances from 8.5 S cm^−1^ at y = 0 to 93.0 S cm^−1^ at y = 0.03. The Hall effect measurement results shown in Figure S6a reveal that the carrier concentration (*n*) increases from 12.2 × 10^20^ to 38.6 × 10^20^ cm^−3^ at 300 K with increasing Se content. Additionally, the mobility (*μ*) first increases from 2.91 cm^2^ V^−1^ s^−1^ at y = 0 to 5.95 cm^2^ V^−1^ s^−1^ at y = 0.01 and then decreases further with increasing y. The synchronous increase in n and μ after Se doping is beneficial for the enhancement in *σ* at RT. To obtain further insight into the mechanism of enhanced *σ* after Se doping, the activation energy of electrical resistivity (E_a_) was estimated by the following Arrhenius equation [40]:

(2) $$\rho =\rho_{0}exp(-E_{a}/k_{B}T)$$

where *ρ* is electrical resistivity, *ρ_0_* is the temperature-dependent constant, and *k_B_* is the Boltzmann constant. The plot of ln *ρ* vs. 1/T in the range of 300 K < T < 423 K has two linear portions with different slopes corresponding to E_a1_ and E_a2_. As shown in Figure 4b and Table S1, E_a1_ and E_a2_ decrease with increasing y, indicating that the Fermi level approaches the valance band and the band structure has been altered [40,41], which contributes to the electrical conduction. To sum up, we deduce that the increase in *σ* can be attributed to the increased content of Cu_1.96_S, which possesses a higher Cu vacancy concentration, and is also simultaneously affected by the movement of the Fermi level. The variation in band structure induced by Se doping can also influence the *μ* of Cu_2−x_S, which will be discussed hereinafter.

The *α* is positive for all samples over the measured temperature range in Figure 4c, demonstrating a p-type conduction of the Cu_2−x_S materials [42]. It increases with the rising temperature, which is insensitive to the phase transition from a monoclinic to hexagonal structure [25] unlike *σ*. In addition, with increasing y, *α* exhibits the opposite trend to *σ*: it decreases from 271.8 to 116.2 μV K^−1^ at RT. We calculated the carrier effective mass (m*) using a single parabolic band (SPB) model based on the measured *α* and *n* values. Figure S6b shows the *α* vs. *n* curves called Pisarenko plots for the Cu_2−x_S_1−y_Se_y_ films at RT (more details can be found in Note S4 of Supplementary Materials). The m* decreases after doping while slightly increases with increasing y, which reflects the variation in the band structure. Since *μ* is inversely proportional to m*, $\mu =q\tau /m^{*}$ (*q* is the electric charge and *τ* is the carrier scattering time), the reduced m* after Se doping favors carrier transport, thereby increasing *μ*. Additionally, the m* of the T-Cu_1.96_S is higher than that of the M-Cu_2_S [33], which is the reason for the increase in m* from 3.09 m_e_ at y = 0.01 to 3.39 m_e_ at y = 0.03.

The Hall effect measurement results of the Cu_2−x_S_0.98_Se_0.02_ film show that *n* and *μ* first increase and then decrease with the increase in temperature (see Figure 4d), which is related to the phase transition and consistent with the change in *σ*. The variation in *α* with temperature is a comprehensive effect of *n*, m*, and temperature.

Owing to enhanced *σ* (69.4 S cm^−1^) and high *α* (147.0 μV K^−1^), the Cu_2−x_S_0.98_Se_0.02_ film exhibits a maximum PF of 150.1 μW m^−1^ K^−2^ at RT, which is ~138% higher than that of the undoped Cu_2−x_S film. The PF increases to 244.5 μW m^−1^ K^−2^ at 403 K. Figure 4f shows a comparison of the PF values at RT of our work and reported Cu_2_S-based bulks [25,26,29,43] and flexible films [30,44,45]. The present PF value is outstanding compared to that of the reported Cu_2_S-based flexible films and comparable with that of Cu_2_S_1−x_Se_x_ bulk (PF = 115.2 μW m^−1^ K^−2^) [26]. However, the value is still lower than that of micro/nano Cu_2−x_S bulk [29], which is mainly due to the higher density of the latter.

Because it is hard to peel off the film from the nylon membrane without destroying it, the κ of the Cu_2−x_S_0.98_Se_0.02_ film was not measured. However, we believe that the κ of this film is low for the following reasons: (1) Cu_2_S has a very low κ of 0.45 W m^−1^ K^−1^ [23]. (2) The film contains grains with sizes from nano to submicron, grain boundaries, and heterointerfaces between Cu_1.96_S and Cu_2_S grains, which can scatter phonons to lower the κ.

**Figure 5:**
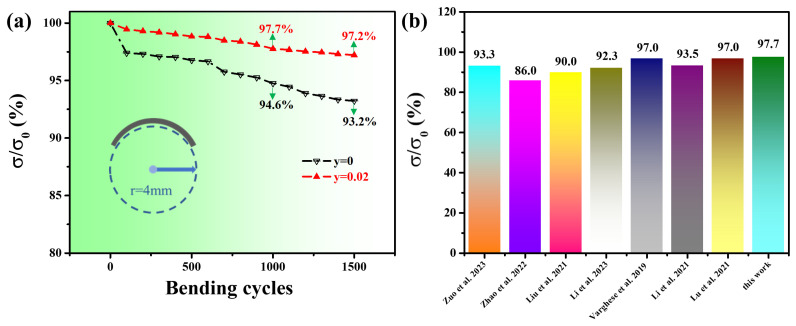
(**a**) Relative electrical conductivity (*σ*/*σ_0_*) as a function of bending cycles for the Cu_2−x_S_1−y_Se_y_ (y = 0 and 0.02) films. (**b**) Comparison of the flexibility of the Cu_2_X (X = S, Se)-, Ag_2_Se-, and Bi_2_Te_3_-based flexible films reported in the literature [14,17,30,44,45,46,47] with a bending radius of 4 mm for 1000 times.

Figure 5a displays the flexibility test result for the Cu_2−x_S_1−y_Se_y_ (y = 0 and 0.02) films. After 1000 and 1500 bending cycles under a bending radius of 4 mm, the *σ* of the Cu_2−x_S_0.98_Se_0.02_ film can retain 97.7% and 97.2% of the initial *σ* (*σ_0_*), respectively, which are higher than that of the Cu_2−x_S film. To understand the enhancement for flexibility, we observed the Cu_2−x_S film and Cu_2−x_S_0.98_Se_0.02_ film both having a similar thickness (~10 μm) by SEM. SEM images (see Figure S7) show that the Cu_2−x_S_0.98_Se_0.02_ film contains fewer pores and is denser than the Cu_2−x_S film. Cracks are generated at pores and fewer pores contribute to the better flexibility. Compared to the *σ*/*σ_0_* results of reported Cu_2_X (X = S, Se) films [30,44,45,46] and other films (Bi_2_Te_3_ [14], Ag_2_Se [17,47]) under the same test conditions (bending radius of 4 mm, bending cycles of 1000 times), the Cu_2−x_S_0.98_Se_0.02_ film exhibits outstanding flexibility, which is mainly due to the excellent flexibility of nylon and the improvement in the density of the film.

To verify the potential application of the Cu_2−x_S_0.98_Se_0.02_ film, a four-leg f-TEG was assembled (see the details on the fabrication process in Note S2 of the Supplementary Materials). The internal resistance (*R_in_*) of the whole device is measured to be ~254 Ω. According to the resistance (*R_1_*~236.4 Ω) of the four legs calculated from the *σ* of the corresponding film, the contact resistance (*R_c_*) is 17.6 Ω if the resistance of the metal electrode is neglected.

**Figure 6:**
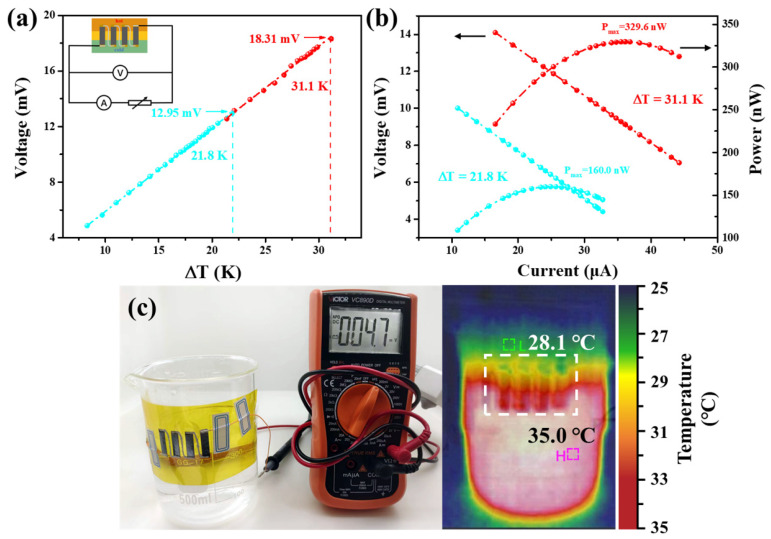
Output performance of the four-leg f-TEG assembled with the Cu_2−x_S_0.98_Se_0.02_ film: (**a**) Open-circuit voltage at different Δ*T*. The inset is a circuit diagram for the output performance test. (**b**) Output voltage and power vs. current at different Δ*T*. (**c**) f-TEG attached to a beaker half-filled with warm water to harvest heat. A voltage of 4.7 mV created by the f-TEG from the Δ*T* across the water surface in the beaker (the corresponding infrared thermal image is on the right, with the Δ*T* of 6.9 K).

Figure 6a shows the open-circuit voltage (*V_oc_*) generated by the f-TEG at different Δ*Ts*. V_oc_ exhibits a primary linear relationship with ΔT, corresponding to the equation *V_oc_ = N∣α∣*Δ*T* (*N* is the number of legs). When the Δ*T* is 21.8 and 31.1 K, *V_oc_* is measured to be 12.95 and 18.31 mV, respectively. Furthermore, the f-TEG was connected into a circuit, as shown in the inset of Figure 6a, to obtain the output characteristics. Figure 6b records the output voltage (*V_out_*) and power (*P_out_*) vs. current by adjusting load resistance (*R_load_*) under different Δ*Ts*. Obviously, the measured *V_out_* is inversely proportional to the current, and the calculated *P_out_* displays a tendency to first increase and then decrease with the increasing current. When Δ*T* of 21.8 and 31.1 K is applied, the maximum power (*P_max_*) generated by the device reaches 160.0 and 329.6 nW, respectively, with a *R_load_* of about 240 Ω. According to the following equation:

(3) $$P_{max}=\frac{V_{out}^{2}}{R_{in}+R_{ex}}$$

where *R_ex_* is the external resistance including *R_load_*, and the resistance of the variable resistor box and the ammeter (*R*_2_, measured to be 15.7 Ω), *P_max_* can be obtained when *R_ex_* equals *R_in_*, which is close to our measurement.

The maximum power density (*PD_max_ = P_max_/N·A*, where A is the cross-sectional area of one leg) of the f-TEG is 1.70 W m^−2^ under a Δ*T* of 31.1 K. To facilitate the comparison of the output performance of different f-TEGs, the normalized *PD_max_* (*PD_max_L*/Δ*T^2^*, where L is the length of one leg) is estimated to be ~35.15 μW m^−1^ K^−2^. As shown in Table S2, this work exhibits better output performance compared with reported Cu_x_A (A = S, Te)-based f-TEGs. However, the value of *PD_max_L/*Δ*T*^2^ is inferior to that of reported Cu_2_Se-based f-TEGs. Figure 6c displays a digital photograph of the f-TEGs attached to a beaker half-filled with warm water to generate power. The f-TEG can generate a voltage of 4.7 mV under a Δ*T* of 6.9 K between ambient environment and warm water, indicating that the f-TEG assembled is feasible as an energy supply device for wearable electronics.

## 4. Conclusions

In summary, we successfully prepared Cu_2−x_S_1−y_Se_y_ flexible composite films on a nylon membrane, which contains submicron grains and nanograins. And the composite films possessed more Cu_1.96_S by adjusting the nominal amount of Se, which is beneficial to the enhancement of *σ*. Meanwhile, the calculation for activation energy E_a_ and the carrier effective mass shows that the electronic structure of Cu_2−x_S can be modulated efficiently via varying Se content, thereby optimizing the electrical properties. Consequently, the maximum power factor reaches 150.1 μW m^−1^ K^−2^ for the Cu_2−x_S_0.98_Se_0.02_ film at room temperature, which is approximately 138% higher than that of the pristine Cu_2−x_S film. In addition, the film possesses superior flexibility: 97.2% of the original electrical conductivity is maintained after 1500 bending cycles with a radius of 4 mm, and it is favorable to application. A four-leg f-TEG assembled with the film can generate a voltage of 18.31 mV and a maximum power of 329.6 nW under a temperature difference of 31.1 K. Our work demonstrates that Se doping in Cu_2−x_S can be an effective strategy for the modulation of phase composition and band structure, thereby developing low-cost and eco-friendly Cu_2−x_S-based flexible TE film with enhanced TE performance.

## Figures and Tables

**Table 1:** The ratio of T-Cu_1.96_S and M-Cu_2_S for the Cu_2−x_S_1−y_Se_y_ (y = 0,0.01,0.02,0.03) films at room temperature.

Phase (mol%)	y = 0	y = 0.01	y = 0.02	y = 0.03
T-Cu_1.96_S	0.28	0.34	0.43	0.46
M-Cu_2_S	0.72	0.66	0.57	0.54
T:M	0.39:1	0.51:1	0.75:1	0.85:1

## Data Availability

Data are contained within the article and Supplementary Materials.

## Supplementary Materials

The following supporting information can be downloaded at: https://www.mdpi.com/article/10.3390/nano14110950/s1, Note S1. Experimental details. Note S2. Assembly of flexible TE generator (f-TEG). Note S3. Characterization and measurement. Note S4. Theoretical calculation details for the Pisarenko plot. Figure S1. Schematic plot demonstrating the preparation process of the Cu_2−x_S_1−y_Se_y_ composite film on a nylon membrane. Figure S2. (a) XRD patterns of Cu_2−x_S_1−y_Se_y_ powder with different nominal content of Se. (b) A typical SEM image of Cu_2−x_S_1−y_Se_y_ powder. Figure S3. XPS spectra of the Cu_2−x_S_0.98_Se_0.02_ film. (a) Survey scan. (b–d) High-resolution scans for Cu 2p, S 2p, and Se 3d, respectively. Figure S4. XPS spectra of Cu for the Cu_2−x_S_1−y_Se_y_ (y=0.01,0.02,0.03) films. Figure S5. EDS mapping of the Cu_2−x_S_1−y_Se_y_ film (y=0.02). Figure S6. (a) Carrier concentrations and mobility, (b) dependence between Seebeck coefficient and carrier concentration of the Cu_2−x_S_1−y_Se_y_ films with different Se doping amounts at 300 K (Se content: y=0,0.01,0.02,0.03). Figure S7. Cross-sectional SEM images of (a) the Cu_2−x_S film, (b) the Cu_2−x_S_0.98_Se_0.02_ film at low magnification. (c,d) The SEM images of corresponding films in (a,b) at high magnification, respectively. Table S1. Activation energies E_a_ calculated from the slopes of the temperature dependences of resistivity. Table S2. Comparison of the output properties of some previously reported CuxA (A=S, Se, Te) based f-TEGs and the present f-TEG. References [30,37,45,46,48,49] are cited in the Supplementary Materials.
