# Supplementary material for: Greatly Enhanced Thermoelectric Performance of Flexible Cu2−xS Composite Film on Nylon by Se Doping
Source: Nanomaterials (Basel). 2024 May 28;14(11):950. doi: 10.3390/nano14110950 (PMC11173826; doi:10.3390/nano14110950)
Supplement: Supplementary file 1 [file nanomaterials-14-00950-s001.zip › nanomaterials-3002907-supplementary.pdf]

Supplementary Materials

Greatly enhanced thermoelectric performance of flexible $Cu_{2-x}S$ composite film on nylon by Se doping

Xinru Zuo, Xiaowen Han, Zixing Wang, Ying Liu, Jiajia Li, Mingcheng Zhang, Changjun Huang and Kefeng Cai*

Key Laboratory of Advanced Civil Engineering Materials of Ministry of Education, School of Materials Science & Engineering, Tongji University, Shanghai, 201804, China

Note S1 Experimental details

Materials

Thiourea (CH_4N_2S)、Se powder and ethanol were bought from Sinopharm Chemical Reagent Co., Ltd., China. $CuCl_2·2H_2O$ (99.9%) was supplied by Aladdin Industrial Corporation. All the reagents were used directly without further purification. A porous nylon membrane with a diameter of 50 mm and pore diameter of 0.22 μm, was purchased from Haiyan Taoyuan Group.

Preparation of $Cu_{2-x}S_{1-y}Se_y$ composite films on nylon membrane

In a typical procedure, a certain amount of Se powder (molar ratio of Se/S is 0.01, 0.02 and 0.03, respectively) was first added into 60 mL of deionized (DI) water under stirring. Then, 3.6 mmol thiourea (Tu) and 7.56 mmol $CuCl_2·2H_2O$ were successively dissolved into the above solution. Afterward, they were sonicated for 30 min and then

transferred into a 100 ml Teflon-lined stainless-steel autoclave at 180 °C for 18 h. After naturally cooling down, the products were washed with DI water and ethanol for 3 times. The as-synthesized powders were ultrasonically dispersed in ethanol and then deposited on a porous nylon membrane through vacuum-assisted filtration. Finally, the $Cu_{2-x}S_{1-y}Se_y$ flexible composite films were obtained after vacuum drying at 60 °C and hot pressing at 270 °C and 1 MPa for 30 min.

Note S2 Assembly of flexible TE generator (f-TEG)

The optimal film was cut into four strips of 2 cm×0.5 cm to assemble a 4-leg f-TEG and the process is as follows: two ends of each strip were coated with a thin layer of Au by evaporation to reduce contact resistance; then the four strips were pasted on a polyimide substrate with an interval of 0.5 cm; finally, Ag paste (SPI#04998-AB) was used to connect the strips in series.

Note S3 Characterization and measurement

X-ray diffraction (XRD, Bruker D8 Advance, Cu K_α radiation, Bruker, Shanghai, China) was performed. Scanning electron microscopy (SEM, Nova NanoSEM 450, Thermo Fisher Scientific, Shanghai, China) and energy-dispersive X-ray spectroscopy (EDS) were used to observe the surface and cross-sectional morphology and composition. X-ray photoelectron spectroscopy (XPS, ESALAB 250Xi Spectrometer Microprobe, Thermo Fisher Scientific, Shanghai, China) was used to analyze the composition and valence states of surface elements. The binding energy was calibrated by setting the standard value of C1s to 284.8 eV. Powder scraped from the $Cu_{2-x}S_{1-y}Se_y$ film was observed by transmission electron microscopy (TEM, JEM-2100F, JEOL,

Shanghai, China), and it was prepared as follows: peel a piece of the film from the nylon membrane, grind the piece with an agate mortar into very fine powder in alcohol, and then ultrasonic treat in alcohol for 30 min, and finally drop a little on a microgrid.

The films were cut into 1.3 cm × 0.3 cm strips to test the temperature dependence of electrical conductivity (σ) and Seebeck coefficient (α) with a TE test system (CTA Cryoall, Beijing Cryoall, Shanghai, China) under the protection of He. The Hall coefficient was measured by the Hall measurement system (LakeShore 8404). σ was measured before and after bending around a 4 mm-radius rod to test flexibility.

The assembled f-TEG was connected with wires into a circuit with a rheostat and a microammeter in series, as shown in the inset of Figure 6a for the output performance test. The hot-end temperature was controlled by heating a copper block ($T + \Delta T$) and the other end was put on an adiabatic foam acting as the cold side (T). The temperature at both ends was measured by two thermocouples. The output voltage and current were collected by adjusting the variable resistor box at a specific ΔT, which was varied by setting different heating temperatures.

Note S4 Theoretical calculation details for the Pisarenko plot

The Pisarenko plot, namely the n-dependent α is obtained based on the single parabolic band (SPB) model.

The expression for "Fermi-Dirac" integral which is associated with α is as follows:

$$F_i(\eta) = \int_0^\infty f\varepsilon^i \, \mathrm{d}\varepsilon = \int_0^\infty \frac{\varepsilon^i}{1+\exp(\varepsilon-\eta)} \mathrm{d}\varepsilon \qquad \text{(S1)},$$

where i is the index of the integral, depends on both the transport property and energy dependence of the relaxation time of the charge carriers. η and ε represent the reduced Fermi level (E_F/k_BT) and the reduced energy (E/k_BT), respectively.

Within SPB model, α can be expressed as follows:

$$\alpha = \frac{k_B}{e}\left(\frac{(5/2+\lambda)F_{3/2+\lambda}}{(3/2+\lambda)F_{1/2+\lambda}} - \eta\right) \quad \text{(S2)},$$

where k_B is Boltzmann's constant, λ stands for scatter factor. Assuming a dominated scattering by acoustic phonons, λ takes on the value of -1/2. Equation S2 can be simplified as :

$$\alpha = \frac{k_B}{e}\left(\frac{2F_1(\eta)}{F_0(\eta)} - \eta\right) \quad \text{(S3)},$$

Electrical conductivity (σ)

$$\sigma = n_H \mu_H e \quad \text{(S4)},$$

Hall carrier concentration (n_H)

$$n_H = \frac{4\pi}{r_H}\left(\frac{2m^* k_B T}{h^2}\right)^{3/2} F_{1/2}(\eta) \quad \text{(S5)},$$

where m^* is the density of states effective mass, h is Planck's constant, r_H is the Hall factor for acoustic phonon scattering, which can be given by

$$r_H = \frac{3}{4}\frac{F_{1/2}(\eta)F_{-1/2}(\eta)}{F_0^2(\eta)} \quad \text{(S6)}.$$

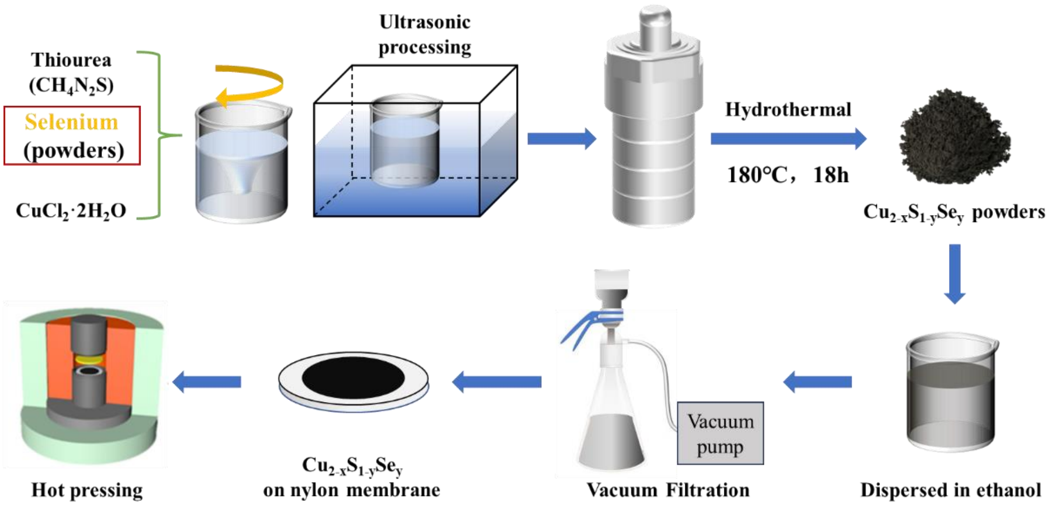

Figure S1. Schematic plot demonstrating the preparation process of the $Cu_{2-x}S_{1-y}Se_y$ composite film on a nylon membrane.

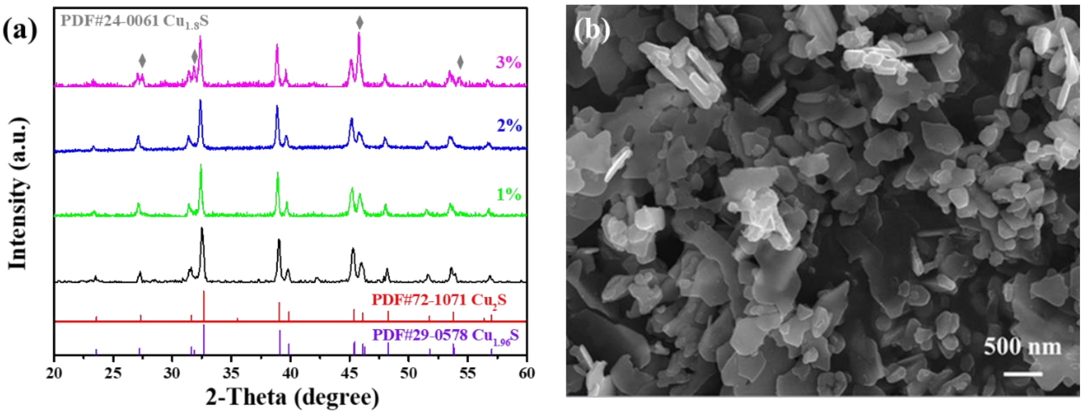

Figure S2. (a) XRD patterns of $Cu_{2-x}S_{1-y}Se_y$ powder with different nominal content of Se. (b) A typical SEM image of $Cu_{2-x}S_{1-y}Se_y$ powder.

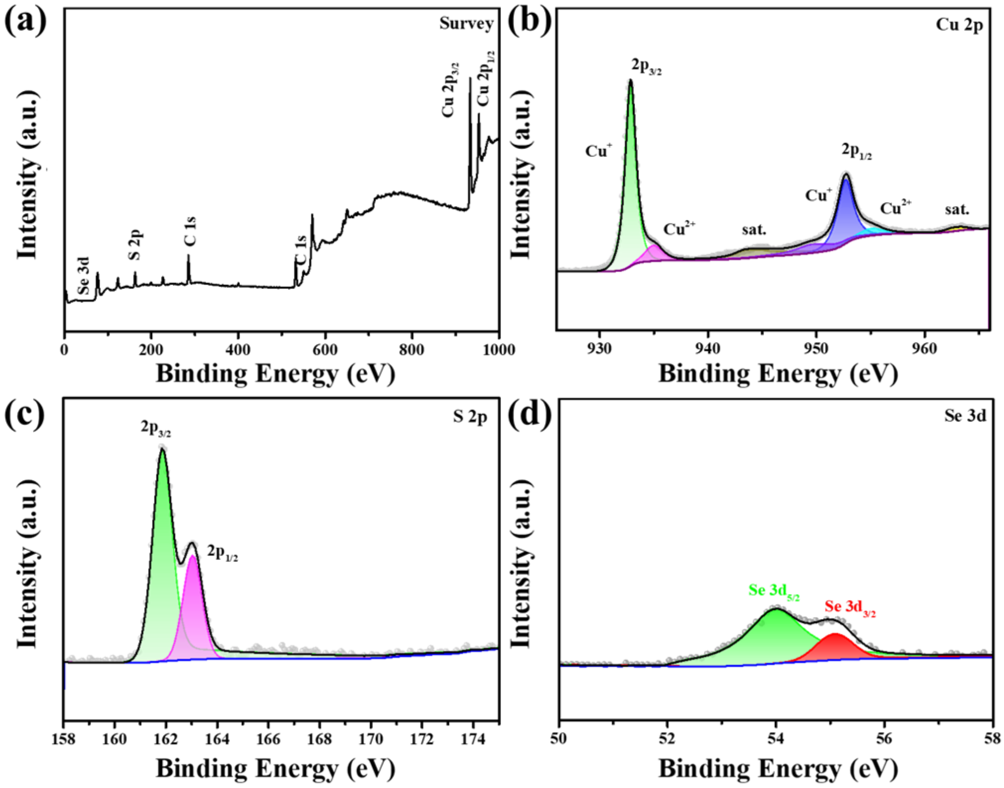

Figure S3. XPS spectra of the $Cu_{2-x}S_{0.98}Se_{0.02}$ film. (a) Survey scan. (b–d) High-resolution scans for Cu 2p, S 2p, and Se 3d, respectively.

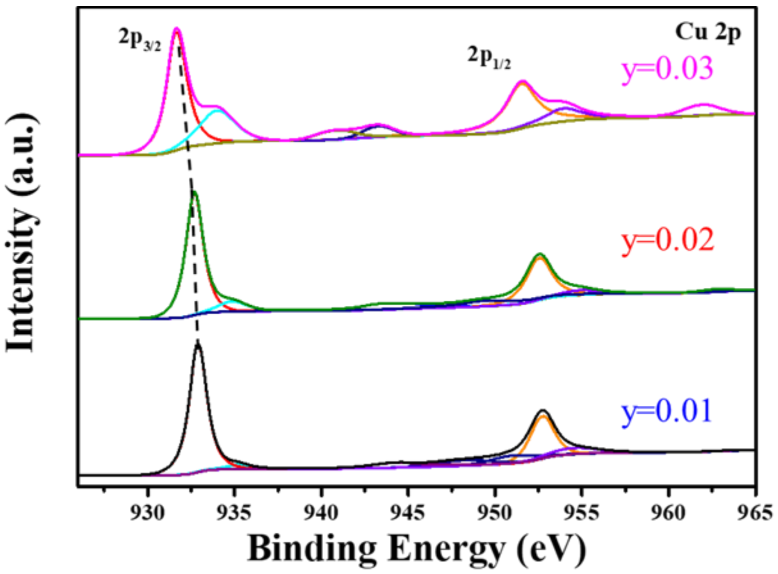

Figure S4. XPS spectra of Cu for the $Cu_{2-x}S_{1-y}Se_y$ (y=0.01,0.02,0.03) films.

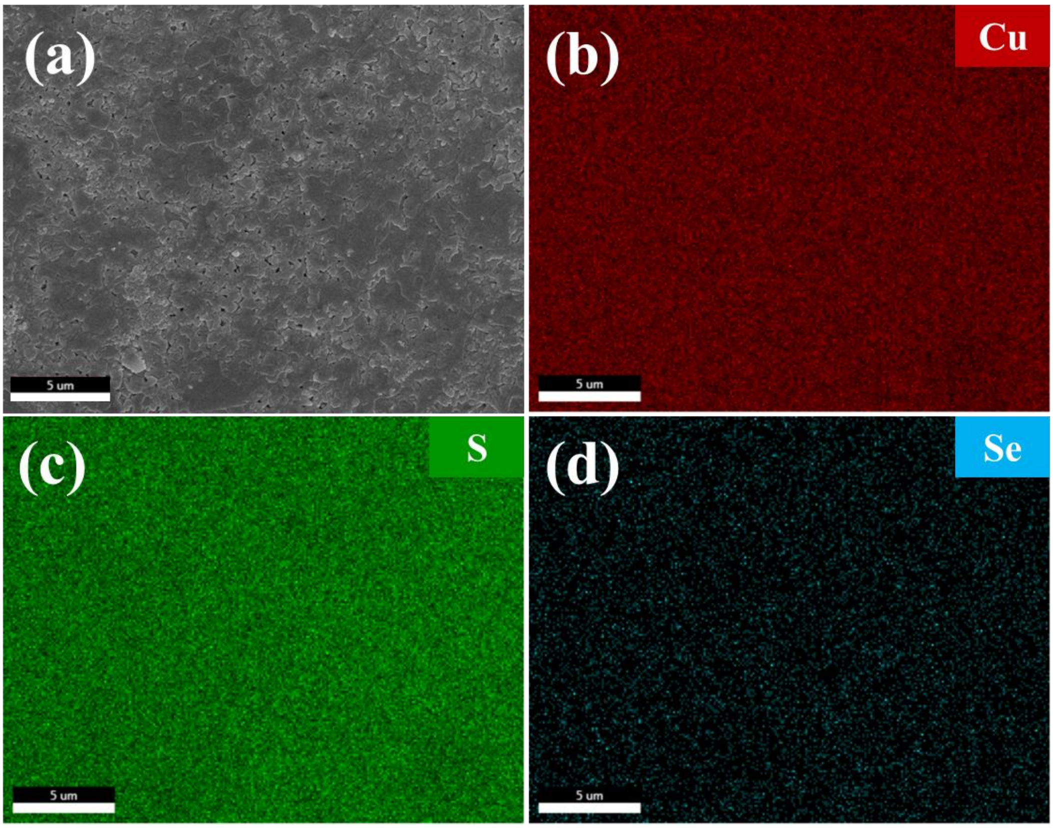

Figure S5. EDS mapping of the $Cu_{2-x}S_{1-y}Se_y$ film (y=0.02).

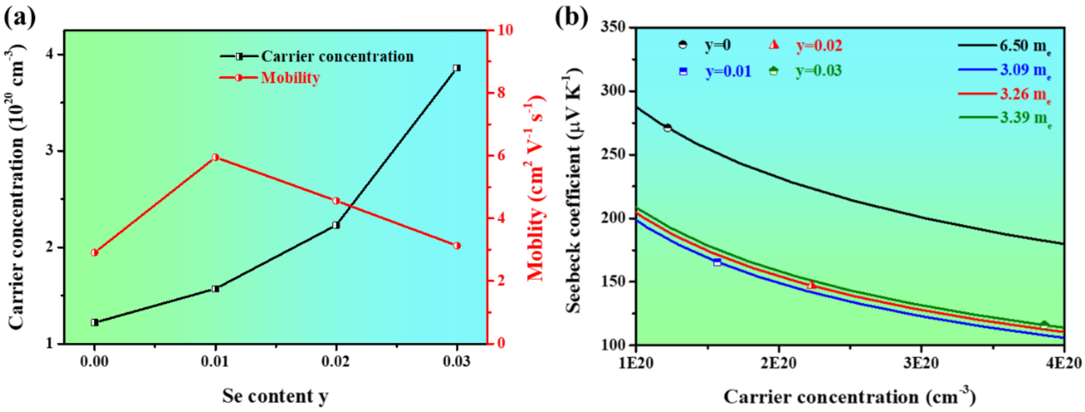

Figure S6. (a) Carrier concentrations and mobility, (b) dependence between Seebeck coefficient and carrier concentration of the $Cu_{2-x}S_{1-y}Se_y$ films with different Se doping amounts at 300 K (Se content: y=0,0.01,0.02,0.03).

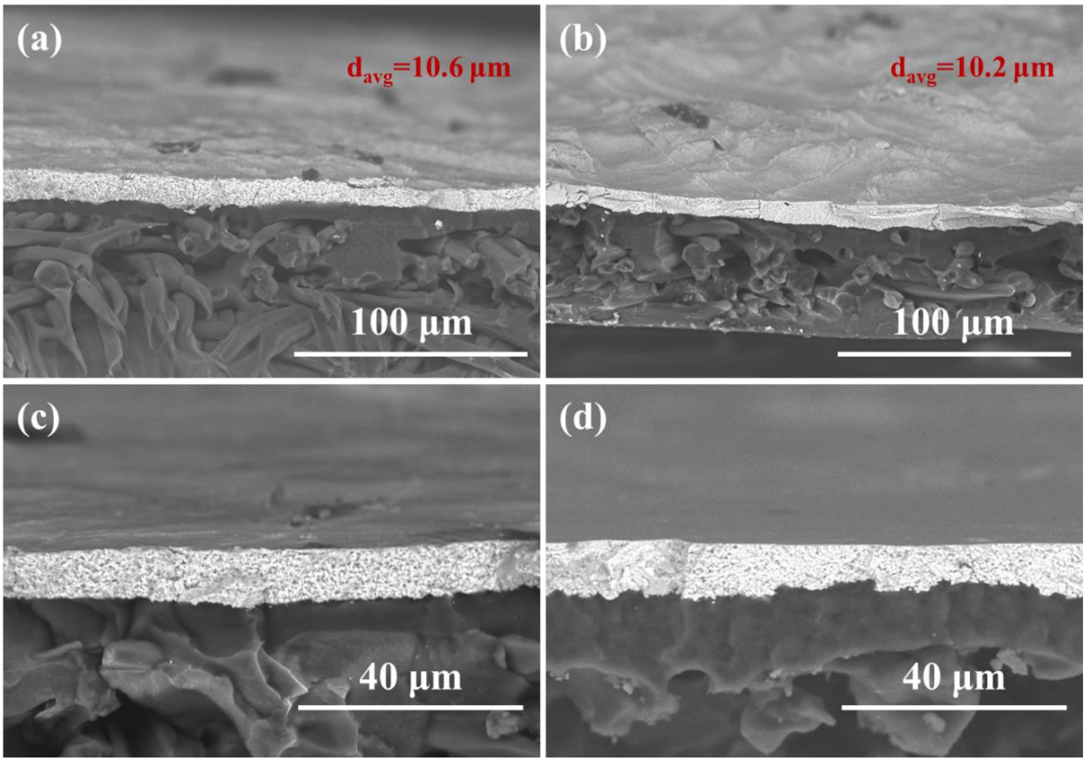

Figure S7. Cross-sectional SEM images of (a) the $Cu_{2-x}S$ film, (b) the $Cu_{2-x}S_{0.98}Se_{0.02}$ film at low magnification. (c, d) The SEM images of corresponding films in (a, b) at high magnification, respectively.

Table S1 Activation energies E_a calculated from the slopes of the temperature dependences of resistivity.

Se content (y)	0	0.01	0.02	0.03
E_{a1}	0.29	0.25	0.22	0.14
E_{a2}	0.19	0.13	0.10	0.09

Table S2 Comparison of the output properties of some previously reported Cu_xA (A=S、Se、Te) based f-TEGs and the present f-TEG.

Materials	Method	N	ΔT (K)	U_{oc} (mV)	P_{max} (nW)	$PD_{max}\cdot l/\Delta T^2$ ($\mu W\ m^{-1}\ K^{-2}$)	Ref.
Cu_2S/PEDOT:PSS	VAF+CP	4	30	2.3	23.06	0.54	[1]
Cu_2Se/PI	MS	10	50	2.7	70	44.5	[2]
$Cu_{2-x}Se$/Nylon	ST+DC	1	6.6	0.2	197	58.4	[3]
Cu_2Se/PEDOT:PSS/N*	WC+VAF	9	30	16	328.0	25.28	[4]
Te/PEDOT:PSS/Cu_7Te_4	WC+DC	8	39.1	31.2	94.7	6.5	[5]
$Cu_{2-x}Mn_yS$/Nylon	HT+HP	4	30.1	16.6	249.5	27.2	[6]
$Cu_{2-x}S_{1-y}Se_y$/Nylon	HT+HP	4	31.3	18.3	329.6	35.2	This work

VAF= Vacuum assisted filtration; CP=Cold Pressing; MS= Magnetron Sputtering; ST=

Solvothermal method; DC=Dip-Coating; WC=Wet-chemical method; HT= Hydrothermal method; HP= Hot Pressing.

References

[1] Liu D, Yan Z, Zhao Y, et al. Facile self-supporting and flexible Cu_2S/PEDOT:PSS composite thermoelectric film with high thermoelectric properties for body energy harvesting [J]. Results in Physics, 2021, 31.

[2] Zheng Z-H, Zhang D-L, Jabar B, et al. Realizing high thermoelectric performance in highly (0l0)-textured flexible Cu_2Se thin film for wearable energy harvesting [J]. Materials Today Physics, 2022, 24.

[3] Li J, Gao J, Jia L, et al. Realization of high output power density in solution-processed flexible α-Cu_2Se film by compositional off-stoichiometry and optimization of length [J]. Applied Surface Science, 2023, 619.

[4] Lu Y, Ding Y, Qiu Y, et al. Good Performance and Flexible PEDOT:PSS/Cu_2Se Nanowire Thermoelectric Composite Films [J]. ACS Applied Materials & Interfaces, 2019, 11(13): 12819-29.

[5] Lu Y, Qiu Y, Jiang Q, et al. Preparation and Characterization of Te/Poly(3,4-ethylenedioxythiophene):Poly(styrenesulfonate)/Cu_7Te_4 Ternary Composite Films for Flexible Thermoelectric Power Generator [J]. ACS Applied Materials & Interfaces, 2018, 10(49): 42310-9.

[6] Zuo X, Han X, Lu Y, et al. Largely Enhanced Thermoelectric Power Factor of Flexible $Cu_{2-x}S$ Film by Doping Mn [J]. Materials, 2023, 16(22).
